# Management of the Positive Axilla in 2017

**DOI:** 10.7759/cureus.1216

**Published:** 2017-05-03

**Authors:** Tolga Ozmen, Alicia Huff Vinyard, Eli Avisar

**Affiliations:** 1 Surgical Oncology, University of Miami, Miller School of Medicine; 2 Department of Surgery, University of Miami, Miller School of Medicine

**Keywords:** axillary dissection, breast cancer, sentinel lymph node biopsy, staging, surgery

## Abstract

The role of surgery in breast cancer treatment and axillary staging is changing. It is not very far from today, when axillary dissection was the standard treatment and staging procedure for all stages of breast cancer. Today, we are on the edge of omitting axillary dissection even in patients with the axillary disease. In this review, we walked through the changes in axillary management throughout the past hundred years.

## Introduction and background

Almost 100 years ago, routine removal of the axillary nodes in what is known as an axillary dissection (AD) became part of the standard surgical treatment of breast cancer after the publication of the autopsy studies performed by the German pathologist Rudolf Virchow’s (1821-1902). Virchow hypothesized that breast cancer had a centrifugal expansion invading first the axillary nodes and spreading to other sites after exceeding axillary nodal station’s loading capacity [1-2]. Soon after, William Stewart Halsted (1852-1922), one of the famous American surgeons at the time, based his "Radical Mastectomy" technique on Virchow's theory and argued that local structures should be extirpated as much as possible to reduce the recurrence risk [3]. From that time on, surgeons treated early stage breast cancer by removing both the whole breast and the axillary contents for almost a century.

## Review

Despite the extensiveness of the surgery, the treatment results were far from being satisfying. One-third of clinically node negative patients had a relapse after MRM and only 18% of breast cancer patients had an annual death rate similar to the age-matched healthy population’s rate [4]. It was obvious that locoregional treatment of the disease was not sufficient for most patients and researchers turned their interest towards studies on systemic therapies. Meanwhile, they also started questioning the extent of surgery for early stage breast cancer. In the 80s, two large studies namely the NSABP-B04 and the King's-Cambridge trials published their 10-year follow-up results after comparing AD plus radiotherapy (RT) with no treatment to the axilla in clinically node-negative patients [5-6]. In both studies, treating the axilla did decrease the recurrence rate significantly (1.4% for AD, 3.1% for RT, and 14% for no treatment), but did not improve the survival rate in early stage breast cancer patients.

The NSABP-B04 trial also reported a 40% metastatic rate on final pathology in clinically node-negative patients but if the axilla did not receive any local treatment only a 15% axillary recurrence was identified [5]. After 1990s with breast cancer screening programs becoming more widely used, the rate of axillary positivity decreased to 22% [7]. AD, however, became an essential staging procedure enabling stratification of the patients to the appropriate adjuvant treatment. This procedure, however, also exposes 78% of early stage breast cancer patients to overtreatment with a 16% risk of lymphedema in five years [8]. With the appearance of the lymphatic mapping technique, three seminal studies, MILAN, NSABP-B32, and ALMANAC trials established sentinel lymph node biopsy (SLNB), a much less invasive procedure, as the new standard for staging of the axilla [9-12]. In all three studies, SLNB was compared to AD for axillary staging in clinically node-negative patients reporting a 5-10% false negative rate (FNR) and 90% identification rate (IR) with equivalent disease-free survival (DFS) and overall survival (OS). Those findings led to the replacement of AD with SLNB for the staging of the clinically negative axilla.

After implementation of SLNB in clinical practice, the quantity of lymph nodes sent to pathology decreased, while the intensity of the pathological examination increased. Utilization of multiple sectioning and immunohistochemical (IHC) staining methods led to upstaging of the axilla and the rate of axillary node positivity increased to 30% [13]. Detection of isolated tumor cells (ITC, tumor invasion size <0.2 mm) and micrometastasis (tumor invasion size >0.2 mm but <2 mm) was the main reason for this increase in axillary positivity. Nevertheless, the implication of this upstaging on survival and recurrence rates was unclear. In a second analysis of the NSABP-B32 trial, previously negative nodes by hematoxylin and eosin (H&E) were retrospectively re-examined using IHC staining [14]. The study reported an occult metastasis rate of 15.9%, with 2/3 of these diagnoses being ITC and 1/3 being micrometastasis. DFS rates for patients with occult metastasis were 94.6% compared to 95.8% for patients with negative nodes. This difference was statistically significant, but clinically not significant. The IBCSG 23-01 and the Spanish AATRM studies prospectively compared completion of AD with SLNB alone in the treatment of patients with micrometastasis and found equivalent results (five-year DFS 87.8% in the "No AD" group vs. 84.4% in the AD group, p = 0.16 in the IBCSG 23-01 trial; one percent recurrence rate in the AD group vs. 2.5% in the "No AD" group, p = 0.325 after five-year follow-up in the AATRM trial) [15-16]. It was recommended by the authors not to implement multiple sectioning and IHC staining methods in the routine examination of sentinel lymph nodes (SLN), since detection of micrometastasis and ITC did not impact the treatment outcomes.

Subsequent to those changes and with improvements in the systemic therapy of breast cancer, researchers started investigating, whether there is a subgroup of patients with macrometastatic disease in the axilla, who could avoid a completion of AD without detriment in their clinical outcome. Two older studies from the 90s, the IBCSG 10-93 study and an Italian study, investigated the need for axillary clearance in elderly patients (over 60-65 years of age) [17-18]. IBCSG 10-93 published their six-year results and the Italian study published their 15-year results. Both studies revealed similar outcomes with and without axillary clearance after long follow-up periods. Most of the patients did not receive SLNB, since SLNB was not a part of the routine clinical practice at that time.

New prospective randomized studies were designed to investigate whether AD can also be omitted for some breast cancer patients with positive SLNB results. ACOSOG-Z0011 and AMAROS trials recruited patients with limited macrometastasis to the axilla [19-20]. ACOSOG-Z0011 reported no additional benefit in regional control of the axilla for completion of AD in this specific group of patients with low recurrence risk (0.9% vs. 0.5%, p > 0.05). The study was however closed early because of low accrual and was heavily criticized for being underpowered. It was also blamed for being unblinded, the reason for which most patients in the observation arm received RT with high breast tangents. AMAROS trial compared treating SLNB positive patients with AD vs. radiation treatment to the axilla. This trial also reported no additional benefit of AD compared to RT in DFS (86.9% in the AD group vs. 82.7% in the RT group, p = 0.18). The POSNOC trial is now recruiting patients with limited axillary disease and will provide more reliable evidence on the comparison of axillary clearance vs. no further surgery to axilla [21].

With the introduction of new chemotherapeutic agents, the success rate of breast cancer treatment improved significantly. Chemotherapy can be administered before (neoadjuvant chemotherapy [NAC]) or after the surgery (adjuvant chemotherapy). Both methods have their own pros and cons. One of the significant benefits of NAC is downstaging of the tumor in the breast and in the axillary nodes and thereby enabling breast conservation and possibly avoiding AD [22-23]. With certain tumor subtypes as triple negative and Her2 (+) breast cancer, axillary pathological complete response (pCR) rates of 30% have been reported when using anthracycline-based regimens reaching 40% with the use of taxane-based regimens [24-25]. In Her2 (+) patients, the addition of trastuzumab increases the pCR rates in the axilla to 70% [26]. In the largest single institutional study from Anderson, the researchers compared performing SLNB before and after NAC [27]. IR and FNR for SLNB before and after NAC were similar (98.7% vs. 97.4% [p = 0.017] and 4.1% vs. 5.9% [p = 0.39], respectively). Multicenter studies also reported concordant results. In a retrospective analysis of NSABP B-27, one of the largest studies comparing NAC to adjuvant therapy, SLNB performed after NAC was reported to be associated with an IR of 85% and an FNR of 11%. In the subgroup analysis, patients, who were clinically positive during the presentation, had a lower FNR in comparison to clinically negative patients (7% vs. 12.4%, p = 0.51). In addition, the use of radionuclide for lymphatic mapping decreased the FNR to nine percent in comparison to 14% when only lymphazurin was used (p = 0.5). In the GANEA study, which prospectively evaluated SLNB followed by confirmatory AD after NAC, the investigators reported an IR of 90% and an FNR of 11.5% [28]. In this study, patients, who did not have a palpable lymph node during presentation had a higher IR in comparison to cN1 patients (94.6% vs. 81.5%, p = 0.008). FNR was also lower in cN0 patients (9.4%) in comparison to cN1 patients (15%), but the difference was not statistically significant (p = 0.66). Combining results from NSABP-B27 and GANEA studies, an IR of 86.5% (lower than a priori SLNB) and an FNR of 10.9% (comparable to a priori SLNB) were found [29]. Two meta-analyses assessed the accuracy of SLNB after NAC. The first study summarized 21 studies/1273 patients and found an IR of 90% and an FNR of 12% [30]. The second study summarized 24 studies (1799 patients) and found an IR of 89.6% and an FNR of 8.4% [31].

Eventually, after those studies, SLNB is usually performed following NAC in cN0 patients by most surgeons. One of the new questions arising when performing SLNB after NAC was how to manage the clinically positive axilla that became clinically negative after NAC. Although the standard of care was to perform an AD, the growing incidence of pCR in the axillary nodes led to the hypothesis that an SLNB could be justified in those patients. The ACOSOG-Z1071 study investigated the validity of SLNB after NAC for patients who were clinically positive before treatment but became clinically negative [32]. In this study, a pCR of 41% was found in the axilla after NAC. FNR was found to be 12.6% but when a dual agent was used, the FNR further decreased to 10.8%. In further subgroup analysis, when two or more SLNs were excised and SLNs were stained using IHC method, FNR was found to be as low as 8.7%. In 1/3 of the patients, a clip was placed into the biopsied lymph node prior to NAC and when the lymph node with clip placement was identified and excised during SLNB, the FNR decreased to 6.8%. In the same group of patients, who had a clip in the biopsied node, the FNR was as high as 39% if the clipped node was not found. In this same study, the significance of a micrometastasis in a sentinel node after NAC was found to be much different than in a non-treated patient. Indeed, further involvement of non-sentinel nodes was found to be present in 97.9% of the cases. The SENTINA study was the second prospective multicenter study investigating SLNB after NAC in patients, who were clinically positive during the presentation [33]. They found an IR of 80.1% and an FNR of 14.2%. When only one SLN was excised, the FNR was increased to 24.3%, while when three or more SLNs were excised the FNR was less than 10%. When a single lymphatic mapping tracer was used, the FNR was found to be 16%, while usage of double tracer decreased the FNR to 8.6%. The most recent prospective multicenter study investigating this topic was the SN-FNAC study [34]. In this study, IHC staining was mandatory. An IR of 87.6% and an FNR of 8.4% was reported. When one SLN was removed, the FNR was 18.2%, while when two SLNs were removed, the FNR was 4.9%. When only radionuclide tracer was used, the FNR was 16%, while usage of dual tracers decreased the FNR further to 5.2%.

These studies were summarized in a meta-analysis. The first one involved 2471 patients and found an IR of 89% and an FNR of 14% [35]. According to the first study, usage of IHC decreased FNR to 8.7%, while without IHC, the FNR was 16%. The second study included 3398 patients and reported a pCR of 39.2%, an IR of 90.9%, and an FNR of 13% [36]. Performing SLNB before NAC has the advantage of staging the axilla without the confounding effect of NAC, but postponing SLNB until completion of NAC, enables some patients to avoid an AD and the associated morbidity. In summary, recent studies demonstrated that SLNB can be safely performed in previously node positive patients (clinically or pathologically proven) who became clinically negative at the completion of NAC. Usage of dual tracer, removal of two SLNs, placement of clip during axillary biopsy and retrieving the clip placed LN during SLNB decreased FNR and increased IR significantly. When performing SLNB after NAC, all sizes of residual disease in the SLNB (macrometastasis, micrometastasis, and ITC) should be treated with completion axillary dissection, since only in two percent of patients is the micrometastasis, the only tumor burden in the axilla. Furthermore, the potential consequence of a false negative axillary staging after NAC might have a possibly negative regional effect on the nodal area but will not have a systemic impact since, in the current NAC practice, all the systemic therapy is usually administered prior to surgery. This, of course, would be different, when SLNB is performed before chemotherapy and when false negativity of SLNB might cause an erroneous down staging and a possible omission of systemic treatment.

The AMAROS trial has similar efficiency with RT as with AD in the treatment of axillary disease. That same question is being addressed for patients with residual axillary positivity on SLNB after NAC. The ALLIANCE A011202 trial (https://clinicaltrials.gov/ct2/show/NCT01901094) is currently recruiting patients with breast cancer staged T1-3/N1/M0 at presentation who became clinically negative after NAC. All patients undergo an SLNB and if SLNB is positive, they are randomized to axillary RT or AD.

Although SLNB is a minimally invasive procedure it is still associated with some morbidity including a risk of lymphedema amounting to five percent at five years [8]. The SOUND trial at the European Institute of Oncology is investigating whether ultrasound staging of the axilla could substitute SLNB. Patients with breast cancer (cT1N0) are undergoing an axillary ultrasound followed by FNA if suspicious nodes are identified. Patients with N0 disease after ultrasound +/- FNA are then randomized to SLNB followed by AD as needed vs. axillary observation [37].

## Conclusions

In conclusion, the role of surgery for axillary staging and for the treatment of minimal disease is changing. Breast surgeons should become familiar with the other emerging modalities in order to comprehensively treat breast cancer.
